# Pliny the Younger advances geochronology

**DOI:** 10.1126/sciadv.aeg2624

**Published:** 2026-09-25

**Authors:** Paul R. Renne, William S. Cassata, Caroline E. J. Hasler, Jack N. Carter, Anthony J. Fuentes, Andrew J. Tholt, Andrea Marzoli

**Affiliations:** ^1^ Berkeley Geochronology Center, 2455 Ridge Rd., Berkeley, CA 94709, USA.; ^2^ Department of Earth and Planetary Science, University of California, Berkeley, CA 94720, USA.; ^3^ Dipartimento Territorio e Sistemi Agro-Forestali, Università di Padova, via dell’Università 16, 35020-Legnaro, Italy.

## Abstract

The eruption of Vesuvius volcano witnessed by Pliny the Younger in 79 CE is dated by the argon-40/argon-39 (^40^Ar/^39^Ar) method to 1938 ± 13 years (SD) before measurement in 2025 CE. Achievement of 0.7% precision and 0.4% accuracy at such a young age marks a precedent for ^40^Ar/^39^Ar dating and signals improved applicability of the method for constraining magma systems’ evolution, including volcanic hazard assessment. Improvements in mass spectrometry, neutron irradiation strategy, and determination of interfering neutron-induced isotopes enable this result. The data serve to determine the decay constant for the radioactive decay of potassium-40 (^40^K) to ^40^Ar that is twice as precise as the value determined by disintegration counting, thus improving the accuracy of ^40^Ar/^39^Ar dating throughout geologic time, and serves as an important datum for intercalibrating ^40^Ar/^39^Ar with other dating methods.

## INTRODUCTION

Accurate and precise dating of prehistoric volcanic eruptions is critical to understanding many aspects of Earth’s evolution. Among the various modes of eruption, pyroclastic flows resulting from large-volume explosive eruptions are of particular interest for several reasons. Such eruptions, typified by sustained columns of ash and gas rising into the stratosphere before collapse, are termed Plinian in homage to Pliny the Younger, who witnessed and described this phenomenon in the 79 CE eruption of Vesuvius. Distal deposits of these eruptions provide unique time markers whose ages are used to calibrate the geological timescale and to infer links between diverse geological phenomena, such as between climate cycles and orbital variations (*1*–*3*), understanding the environmental effects of volcanism (*4*) and constraining the causes of mass extinctions over geologic time (*5*).

Pyroclastic flows from Plinian eruptions pose exceptional risk to nearby human populations, causing an estimated 150,000 mortalities from 18 such eruptions worldwide in the past 2000 years (*6*). Today, tens of millions of people are at risk from pyroclastic eruptions of historically active volcanos near densely populated areas including Naples (Italy), Yogyakarta (Indonesia), and Mexico City (Mexico). Dating such events in the younger range of prehistory is important for assessing long-term volcanic hazards. Adequately precise eruption ages can be used to establish recurrence intervals, which inform physics-based volcano monitoring that may enable short-term eruption forecasting (*6*, *7*). The importance of dating eruptions for volcanic hazard assessment is exemplified by the recent documentation of a caldera-forming eruption at 29 thousand years (ka) (*8*) from the Campi Flegrei magma system encompassing Naples, which occurred between previously documented super-eruptions at 40 ka (*9*) and 15 ka (*10*). This discovery, “has drastically reduced the expected repose interval for caldera-forming eruptions at Campi Flegrei” (*11*).

In addition to its implications for volcanic hazards, high-precision geochronology enables researchers to infer magma system properties including magma residence times, recharge, and eruptive behavior over short geological timescales (*2*). Volcanic deposits dated accurately and precisely by one method also provide critical benchmarks for testing and refining other dating techniques (*1*, *12*, *13*).

One of the most important methods of dating volcanic eruptions is the ^40^Ar/^39^Ar technique. Like most radioisotope-based methods except radiocarbon, the relative precision of ^40^Ar/^39^Ar dating increases with the eruption’s age, with all other factors being equal. Thus, the method has larger relative uncertainties at younger ages. The youngest ^40^Ar/^39^Ar ages yet published to our knowledge are for lavas that erupted 510 ± 180, 550 ± 120, and 1640 ± 370 years ago (uncertainties here and throughout are 1 SD unless otherwise stated) on Ascension Island (*14*). These were challenging samples lacking separable K-rich minerals, and, despite the large relative uncertainties (>22%), these results were useful in classifying the island as an active volcanic system. The youngest ^40^Ar/^39^Ar ages that can be compared with historical records are for the 79 CE Plinian eruption of Vesuvius, which destroyed the cities of Pompeii and Herculaneum. These measured ages (*15*, *16*) as reported agree with the historically recorded age within their ∼5% relative uncertainties (∼100 years) at 68% confidence, although both are less precise when corrected for overlooked factors and updated calibrations (see the Supplementary Materials). Thus, the historical records serve to validate the ^40^Ar/^39^Ar ages, albeit at relatively low precision. The eruption is widely cited as having begun on 24 August and lasted for ∼2 days, although some authors argue that it occurred 1 or 2 months later in the same year (*17*). The basis for the 79 CE calendar age is somewhat complex; hence, we reviewed the records as summarized in Results.

Other dating methods, (U+Th)/He (*18*) and ^14^C (*19*), have also produced ages indistinguishable from the historical 79 CE age, but at substantially lower precision than the ^40^Ar/^39^Ar results. Tephra from the 79 CE eruption has been tentatively identified in a Greenland (GRIP) ice core at depths corresponding to 79 to 80 CE based on an age model using multiparameter annual layer counting with an estimated uncertainty of ±0.5% (*20*). While the chemical composition of glass shards in this material is consistent with 79 CE Vesuvius tephra, two eruptions in the Azores with comparable estimated volcanic explosivity indices (*21*) of 4 to 5, or unrecognized coeval eruptions elsewhere cannot be definitively ruled out as the source.

When dating volcanic eruptions, the standards of precision and accuracy depend on the questions being asked. To establish eruptive histories that can help to forecast future hazards, dating methods should be as precise and accurate as possible. Dating past eruptions is trivial if reliable historical records are available, but, for prehistoric eruptions, researchers must rely on other methods such as ^40^Ar/^39^Ar dating. Obtaining precise ^40^Ar/^39^Ar ages is especially challenging for relatively young eruptions just beyond the limits of historical records, which is inconvenient because relatively recent eruptions are the most critical for evaluating future eruption probabilities. Because Vesuvius’ 79 CE eruption is a near ideal case for ^40^Ar/^39^Ar dating at the limit of historical records, significant improvement over the existing ∼100 year uncertainty is clearly desirable.

Here, we report new ^40^Ar/^39^Ar dating of the 79 CE Vesuvius eruption with greater accuracy and an order of magnitude better precision than previous work using this or any other method aside from historical records. This result is achieved through improved mass spectrometry and experimental design, and the most definitive measurement of an important interfering isotope correction yet determined. Besides establishing a new benchmark for dating young pyroclastic eruptions, we use the results to derive an improved estimate of the rate constant for the radioactive decay of ^40^K to ^40^Ar.

## RESULTS

### Historical dating of the 79 CE eruption

Because the date of the 79 CE Vesuvius eruption is constrained by historical records, it can serve as a benchmark for radiosotopic dating methods. However, historical and archeological misunderstandings have created confusion surrounding the eruption date, e.g., (*22*). We therefore review the evidence for the date of the eruption.

Reconstructions of the 79 CE Vesuvius eruption rely on two letters written by Pliny the Younger, which provide an eyewitness account of the event (*23*)*.* At the time of the eruption, Pliny the Younger was 17 years old. His letters are often cited as a source for the eruption date. Actually, the letters only record the day and month (24 August), but not the year of the eruption. The year must be inferred from other sources.

The earliest surviving mention of the year comes from Lucius Cassius Dio (early third century CE). Dio’s original text does not survive but is preserved in later paraphrases by medieval historians. Cassius Dio associates the eruption with achievements of the emperor Titus: “As a result of these events in Britain, Titus received the title of *imperator* for the fifteenth time. […] In Campania remarkable and frightful occurrences took place, for a great fire suddenly flared up at the very end of the summer” (*24*). Thus, we can identify the year of the eruption (the “great fire”) by dating Titus’s 15th acclamation as *imperator*, an honorific granted to military commanders.

In ancient Rome, years were identified by significant happenings or by the names of the two consuls—the Roman Republic’s highest elected officials, elected annually—in office at the time. Cassius Dio records that Titus received his 15th *imperator* title the same year his father, the emperor Vespasian, died. According to the Roman historian Suetonius, Vespasian was born “in the consulate of Quintus Sulpicius Camerinus and Gaius Poppaeus Sabinus” and died “at the age of sixty-nine years, seven months and seven days” (*25*). We can therefore calculate the year of Titus’s 15th *imperator* title if we know when Q. S. Camerinus and G. P. Sabinus were consuls.

The conversion from consular to Common Era dates (BCE/CE) was defined by the sixth-century monk Dionysius Exiguus in 525 CE, who anchored his calendar to consular years (*26*). Using Dionysius Exiguus’s system, earlier consular dates can be converted to the modern calendar. This places the consulship of Q. S. Camerinus and G. P. Sabinus in 9 CE and, by extension, the death of Vespasian in 79 CE. As Titus’s 15th acclamation occurred in the same year, the Vesuvius eruption can be confidently assigned to 79 CE.

What about the month and day of the eruption? Pliny writes that the eruption began on the ninth day before the *kalends* of September (*non. kal. Septembres*) or 24 August. This is consistent with Cassius Dio’s account, which suggests an eruption date at the end of summer. Dio’s translators tend to render the relevant passage as “right at the end of summer”. This agrees with the ancient Roman definition of the start of autumn, which was typically placed in early August [e.g., (*27*)].

Despite Pliny’s and Dio’s testimony, the 24 August eruption date has been contested. Archeological finds are sometimes used to support an eruption date in late autumn. In the late 18th century, Carlo Maria Rosini proposed an autumn eruption on the basis of archaeological finds at Herculaneum—braziers, carpets, and fruits typically harvested later in the year, including chestnuts, dates, figs, and raisins (*28*)*.* But other archeologists and historians have pointed out that braziers, autumn fruits, and warm clothing worn by victims do not preclude an August eruption date (*22*). In 2018, a charcoal graffito beginning *xvi k nov* (17 October) was found on a wall in Pompeii (*29*). The graffito has been used to argue that Vesuvius could not have erupted before October because charcoal writing is too fragile to survive many months in the open. However, an experiment conducted in Pompeii showed that charcoal graffiti can easily last from 17 October until 24 August the following year (*30*).

Proponents of a late autumn eruption sometimes refer to a coin hoard found in Pompeii’s “House of the Golden Bracelet”. One of the coins was once thought to reference Titus’s 15th *imperator* title, which was not granted until after 8 September 79 CE. The coin found in Pompeii was initially interpreted to read *IMP XV*, implying that it was minted after that date and precludes a 24 August eruption (*31*). However, a recent reevaluation found that the coin, which is in poor condition, had been misinterpreted and instead reads *IMP XIII* (*32*)*.* Thus, the coin may have been minted before 8 September and can neither confirm nor refute an August eruption date.

We conclude that, although later reinterpretations have proposed autumn or even November dates, there is no solid reason to doubt Pliny’s and Cassius Dio’s accounts. The best available evidence points to 24 August 79 CE as the date of the Vesuvius eruption, and that is the date that we use as a reference point. A more detailed discussion of ancient Roman chronology and evidence supporting the August eruption date can be found in the Supplementary Materials.

### ^40^Ar/^39^Ar geochronology

Data obtained from the Vesuvius 79 CE AM1 sanidine grains extracted from 8 of 10 irradiation wells are shown on an inverse isochron diagram (^36^Ar/^40^Ar versus ^39^Ar/^40^Ar) in Fig. 1. Grains from two irradiation wells (lab nos. 39209 and 39210; see the Supplementary Materials) were excluded because of contamination by older material. Data from these samples yielded ages ranging from within uncertainty of 79 CE to over 20 ka. Aside from samples derived from the two contaminated irradiation wells, all 153 incremental heating steps from all aliquots are colinear on the inverse isochron diagram (Fig. 1). Given that each irradiation well was filled with material from the same prepared sanidine mineral separate, it appears the two irradiation wells that we excluded from the isochron were contaminated during the loading or unloading process. The similarity in K/Ca between the contaminated and uncontaminated (table S1) aliquots suggests that dust or fine grains from adjacent coirradiated 1.18-million-year (Ma) Alder Creek sanidine (ACs), which yield indistinguishable K/Ca ratios, were the source of contamination. The remaining 153 analyses, collectively derived from ∼700 mg of sanidine, define an isochron (Fig. 1) with an age of 1938 ± 10 years, a trapped ^40^Ar/^36^Ar of 299.64 ± 0.24, and a mean squared weighted deviation (MSWD) (*33*) of 1.5 calculated using IsoplotR (*34*). The percentage of ^40^Ar that is radiogenic (%^40^Ar*) as distinct from atmospheric or nucleogenic in individual steps is as high as 43.9% (table S1), a key factor in the precision of the isochron. To account for the excess scatter indicated by the MSWD > 1, we expand the uncertainty by a factor of $\sqrt{\text{MSWD}}$, which yields ±13 years. Thus, the calendar age that we determine is 87 ± 13 CE, indistinguishable at the 1σ level from the historically determined age of 79 CE. A useful metric to represent this result in terms of actual measurements is the *R* value, defined as the ^40^Ar*/^39^Ar_K_ of a sample relative to that of a standard. Accordingly, $R_{\text{ACs}}^{\text{AM}1}$, the *R* value of AM1 relative to that of the ACs standard, is (1.6359 ± 0.0110) × 10^−3^.

**Fig. 1.**
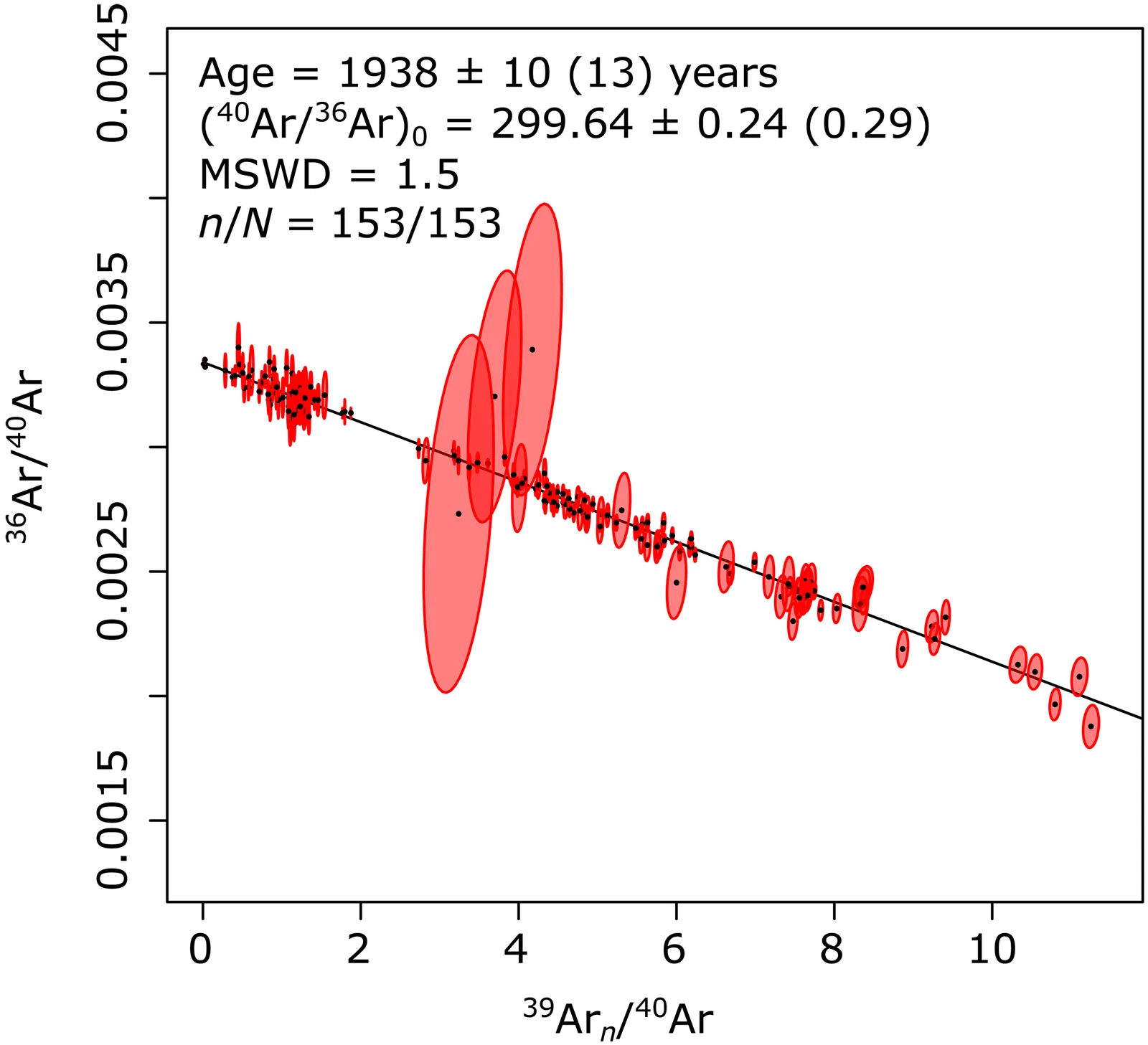
Inverse isochron diagram obtained from of the incremental degassing of 48 aliquots of Vesuvius AM1 sanidine collected near Pompeii. Ellipses denote 1 SD uncertainties, with rotation reflecting uncertainty correlations. The data were regressed with and the figure was modified from IsoplotR (*34*). The numbers in parentheses that follow uncertainties represent the expanded 1 SD uncertainties. Subscript *n* in ^39^Ar*_n_*/^40^Ar indicates values normalized to a uniform *J* value.

The trapped ^40^Ar/^36^Ar derived from the isochron is distinct at 95% confidence from the atmospheric value of 298.56 ± 0.31 (*35*), which argues against calculating ^40^Ar/^39^Ar ages assuming that value (and ignoring its uncertainty, as is common practice). The supra-atmospheric ratio suggests a minor component of magmatic excess ^40^Ar, as was reported in a previous study (*15*).

## DISCUSSION

### Key factors in precision and accuracy

Reproducing the known age of a <2000-year-old eruption by ^40^Ar/^39^Ar methods within ∼0.7% relative uncertainty is unprecedented and portends substantial improvement in the resolution of geologic time with this technique, especially in the late Quaternary Period. The specific factors that enable this improvement include the following:

1) The abundance of the K-rich mineral sanidine is a crucial requirement. Because silicic pyroclastic eruptions tend to be K-rich, sanidine is a common phenocryst in such deposits. In the chemically zoned deposits of the 79 CE Vesuvius eruption, the dated sanidine from the lower white pumice unit exhibits an extremely homogeneous K/Ca that was plausibly in equilibrium with the erupted magma, hence unlikely to contain xenocrysts or antecrysts (*36*).

2) Incremental heating with a laser (cf resistance furnace) enables low atmospheric backgrounds and relatively efficient progressive degassing of trapped (nonradiogenic) argon, allowing the measurement of radiogenic argon exceeding 40% at higher laser power. The resulting spread of data on an isochron and the large number of analyses enables detection of and correction for a slightly supra-atmospheric component.

3) Multicollector mass spectrometry enables high throughput and minimizes uncertainties associated with extrapolating ion beam intensities measured by peak hopping to a common time. ATONA detectors (*37*) enable precise measurement of low-intensity but high-stability ion beams for ^40^Ar and ^39^Ar.

4) Minimal duration (6 min) of irradiation time, cadmium shielding, and precise determination of both (i) the ^40^Ar produced from K by neutron irradiation, which reduces the correction for this interfering reaction to 20 years, with 1 year of uncertainty, and (ii) the production of ^36^Ar from potassium that allows for more accurate corrections for nonradiogenic ^40^Ar (*38*).

5) Rigorous accounting for spatial variations in neutron fluence during irradiation by closely bracketing the unknowns with standards, which reduces the uncertainty associated with the *J* values to <0.1%.

### Broader implications

This study was motivated by a desire to provide additional constraints (priors) to the recently proposed Bayesian calibration of the K-Ar radioisotopic system (*39*), which can be readily expanded to incorporate and jointly intercalibrate additional geochronometers to the K-Ar and U-Pb radioisotopic systems. Radiocarbon (^14^C) dating, in particular, could benefit from improved ^40^Ar/^39^Ar geochronology applied to events within the method’s range of applicability (the past ∼55 ka). Because atmospheric ^14^C/^12^C has varied over time, radiocarbon dates must be calibrated by other means and different calibrations have been developed for different reservoirs (e.g., marine versus nonmarine) and hemispheres (Southern versus Northern). ^40^Ar/^39^Ar dating is an excellent calibrant as it extends past the tree-ring record and can be applied to volcanic deposits that contain charred woody materials that are suitable for ^14^C dating, e.g., (*9*). The radiocarbon community routinely assimilates new calibration data and has developed extensive computational tools for that application, e.g., (*40*). Further, because a given measured ^14^C/^12^C ratio can correspond to multiple ages [(*40*) and references therein], improved ^40^Ar/^39^Ar ages with precision on par with or better than radiocarbon ages (i.e., decadal-scale uncertainties) could resolve degenerate ages. Comparison with other volcanic eruptions whose ages are reliably constrained by historical records could further advance intercalibrations between ^40^Ar/^39^Ar, radiocarbon, and other dating methods, specifically through the improved determination of the ^40^K decay constant provided by the present study.

A noteworthy case that may ultimately contribute to intercalibrating the ^14^C with K-Ar and other radioisotopic systems is the so-called Millenium Eruption (ME) of Changbaishan volcano, which straddles the China-North Korea border. The eruption was dated by ^40^Ar/^39^Ar at 1240 ± 205 years (864 ± 205 CE) (*41*), while ^14^C and various proxy records led to an inferred age of 946 CE (*42*) (no uncertainty reported). Unfortunately, the ^40^Ar/^39^Ar data of (*41*) reveal a large proportion of anomalously old apparent age steps, attributed to excess ^40^Ar, such that 20 of the 56 steps were rejected from the isochron. Anomalously old ^40^Ar/^39^Ar ages for the ME were also reported in another study (*43*) that attributed the results to xenocrystic contamination and concluded that no eruption age could be determined from their data. Despite these documented challenges to ^40^Ar/^39^Ar dating of the ME, further effort could conceivably yield useful results.

### Improved determination of the ^40^K to ^40^Ar decay constant

Beyond demonstrating the temporally extended capability of the ^40^Ar/^39^Ar method, our results presented here foreshadow improved calibration of the method over its entire range of applicability. Our data represent a controlled ingrowth experiment from which the partial decay constant for ^40^K decay to ^40^Ar via electron capture and positron emission can be determined with subpercent uncertainty as described below. Now, the best available experimental determination of this parameter is from liquid scintillation counting and has a relative uncertainty of 1.4% (*44*). A more precise value was inferred from a recent Bayesian intercalibration of the K-Ar and U-Pb systems (*39*). However, the underlying U-Pb decay constants in ubiquitous use are based on a single study (*45*) with poorly quantified systematic uncertainties (*46*). Thus, improved constraints on the ^40^K decay scheme and, in particular, the branching ratio of ^40^K decay to ^40^Ar could significantly improve the absolute calibration of both the K-Ar and U-Pb decay schemes.

Our Vesuvius data allow for an even more precise intercalibration. The standard K-Ar age equation is

t=1λtotln(1+λtotλEC,β+·Ar40∗K40) (1)

where λ_tot_ is the total decay constant and λ_EC,β+_ is the partial decay constant for ^40^K decay to radiogenic ^40^Ar (^40^Ar^*^), which includes electron capture to both an excited and ground state and positron emission. For ingrowth periods that are short compared to the half-life of ^40^K, $\lambda_{\beta -}$ can be neglected and $\left(e^{\lambda_{\text{tot}}t}-1\right)\approx \lambda_{\text{tot}}t$. For a ∼2000-year-old sample like Vesuvius AM1, this approximation is accurate at the ppm level. Equation 1 can thus be simplified and rearranged in terms λ_EC,β+_ as follows

λEC,β+≈(Ar40∗K40)AM1·1t (2)

For the Vesuvius AM1 samples, *t* is known from historical records and (^40^Ar*/^40^K)_AM1_ can be inferred by intercalibrating the Vesuvius AM1 sanidine data to various K-Ar standards for which the concentrations of ^40^K and ^40^Ar* have been determined by first principles. There are four such K-Ar standards: GA-1550, GHC-305, Hb3gr, and NL-25, which have been intercalibrated by ^40^Ar/^39^Ar with the neutron fluence monitor Fish Canyon sanidine (FCs), enabling determination of ^40^Ar*/^40^K = (1.6407 ± 0.0047) × 10^−3^ for FCs (*47*). Using an updated value of ^40^K/K (*48*), this value becomes ^40^Ar*/^40^K = (1.6413 ± 0.0047) × 10^−3^ for FCs. From this, we can determine ^40^Ar*/^40^K of the Vesuvius AM1 sanidine as follows

(Ar40∗K40)AM1=RFCsAM1·(Ar40∗K40)FCs=RACsAM1·RFCsACs·(Ar40∗K40)FCs (3)

where $R_{\text{ACs}}^{\text{AM}1}$ is determined in this study and $R_{\text{FCs}}^{\text{ACs}}$ is from (*49*). The resulting ^40^Ar*/^40^K of Vesuvius AM1 sanidine is (1.1195 ± 0.0081) × 10^−7^. Taking the midpoint of the analytical session (1 June 2025) as the analysis time with a 1-month uncertainty to encompasses the analytical window and an eruptive date of 24 August 79 CE with an assumed 2-month uncertainty to account for vagaries in the historical records (see the Supplementary Materials) yields a total ingrowth time of 1945.77 ± 0.12 years (0.01%).

Solving Eq. 2 using the above values for *t* and ^40^Ar*/^40^K yields λ_EC,β+_ = (5.7551 ± 0.0422) × 10^−11^. This is indistinguishable from the partial decay constant reported by Kossert *et al.* (*44*), but with almost two times higher precision (Fig. 2). For completeness, we also explored the full posterior of λ_EC,β+_ using a parallelized affine-invariant Monte Carlo sampler (*50*). Both pathways converge on the same result (see the Supplementary Materials).

**Fig. 2.**
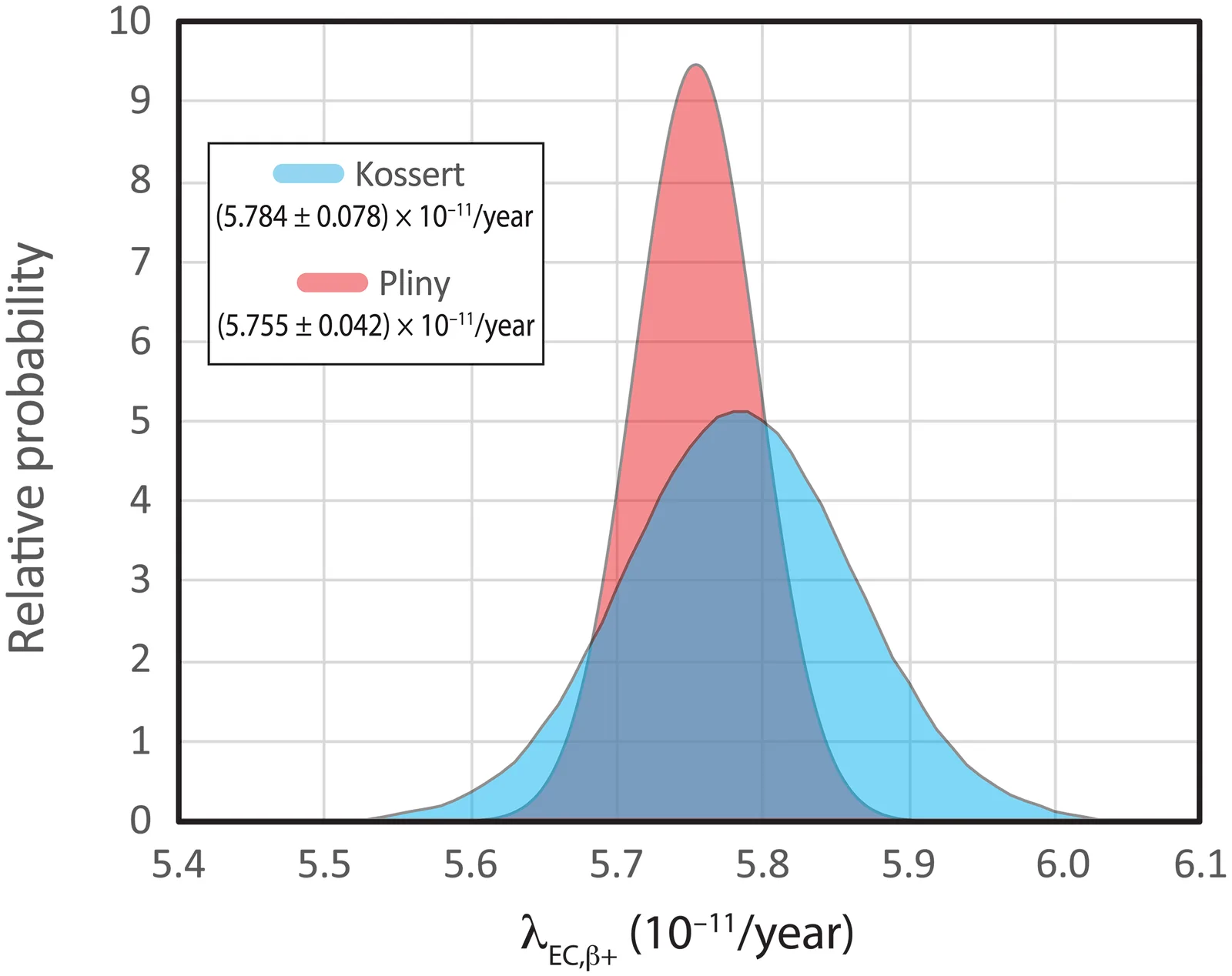
Comparison of the partial decay constant for production of radiogenic ^40^Ar from ^40^K, λ_EC,β+_ from an independent experimental determination and this work. Blue Gaussian shows the experimentally determined λ_EC,β+_ value of Kossert *et al.* (*44*); red Gaussian labeled Pliny shows the λ_EC,β*+*_ inferred in this work from the Vesuvius calibration. The means ± SD is shown for each estimate.

## MATERIALS AND METHODS

Sanidine was separated from ∼2 kg of 2- to 5-cm pumice lapilli collected as sample AM1 at the Villa Poppea, Oplontis, ∼1.8 km WNW of Pompeii. The sampled unit corresponds to the widespread EU2 white pumice fall layer of Cioni *et al.* (*51*), which contains the highest sanidine concentration and most K-rich sanidine of any of the erupted units comprising the stratigraphically zoned sequence recognized here and elsewhere (*51*). The lapilli were crushed with a mortar and pestle, rinsed ultrasonically in 5% HF solution for 5 min, and then repeatedly rinsed ultrasonically with distilled water. After drying overnight at 110°C in an oven, samples were sieved to obtain a 0.71- to 0.42-mm-size fraction, from which pure sanidine crystals devoid of perceptible inclusions or adhesions were handpicked. Fifty grains were analyzed quantitatively by scanning electron microscopy (*36*) and proved to be unzoned as reflected in backscattered electron images (Fig. 3). Note that the bright curvilinear features in the BSE images are artifacts of cracks and voids as seen in the SE images, rather than compositional anomalies.

**Fig. 3.**
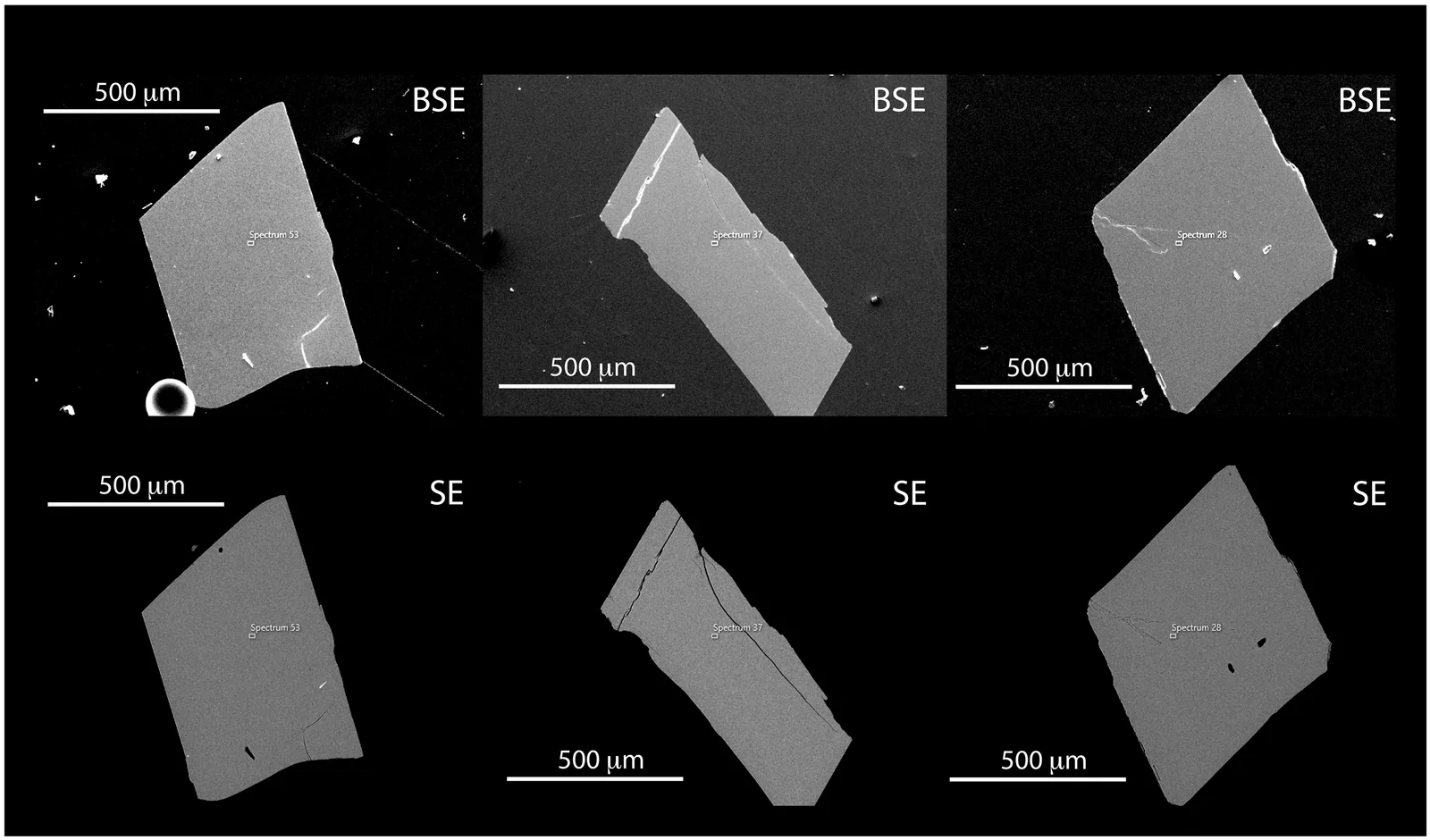
Backscattered electron (BSE; top row) and corresponding secondary electron (SE; bottom row) images for three grains of AM1 sanidine.

Sample grains were loaded into wells in two Al discs (see the Supplementary Materials) interspersed with wells containing the 1.18-Ma ACs standard (*49*) with an age recalculated per Carter *et al.* (*39*), in the 0.84- to 0.42-mm-size fraction. The wells of ACs and AM1 each contained an estimated 16 and 87 mg of sanidine, respectively. The two discs were irradiated for 6 min in the Cadmium-Lined Irradiation Tube of the Oregon State University TRIGA reactor on 28 April 2025. Analyses of the ACs and AM1 were completed between 14 May and 17 June 2025. Gas extraction was by total fusion (ACs) or step heating (AM1) with a 55-W CO_2_ laser. ACs was degassed by total fusion of five grains at a time, with four to seven aliquots per irradiation well. AM1 was degassed by step heating in three to four steps at laser power between 1 and 30 W. The laser beam diameter was 3.0 and 4.5 mm for the ACs and AM1 samples, respectively. Each aliquot of AM1 comprised an estimated 144 grains of sanidine. The neutron fluence parameter *J* was determined for each well containing ACs, with average relative precision of 0.154%. Spatial variation of *J* is evident in both irradiation disks but does not conform to a simple geometry; hence, *J* values for each well containing AM1 were determined as the inverse variance weighted average of values from the three wells of ACs most closely bracketing it, which produced average relative precision of 0.07% for all *J* values.

Following extraction, the released gas was purified by exposure to two SAES GP-50 getters: one at room temperature and one operated at 14.7 V and 1.55 A. Isotopic abundances were measured using an Isotopx NGX mass spectrometer equipped with nine Faraday detectors fitted with ATONA amplifiers and one discrete dynode electron multiplier. All isotopes were measured on the Faraday detectors, except ^36^Ar (see the Supplementary Materials). Signals on the ATONA amplifiers were integrated for 20 s. Total counting durations were 1800 s. Detector baselines were measured at the end of each run as signals at off-peak masses (0.4 u above and below peak center) for 200 s. These baselines were fitted on a time-dependent basis and subtracted from all measured signals. Baseline uncertainties were not explicitly propagated into isotopic measurement uncertainties as background corrections already include noise arising from variations in detector baselines. Background analyses were interspersed with the sample heating steps at typical frequency of one per sample heating step.

A second series of experiments were conducted on K-rich synthetic glass to obtain the (^40^Ar/^39^Ar)_K_ and (^36^Cl/^39^Ar)_K_ needed to correct the data as described in the Supplementary Materials. Additional nuclear interferences, with much smaller effect on the computed ages, are listed in table S1. All Ar isotope data corrected for baselines, backgrounds, radioactive decay, mass discrimination, detector intercalibration, and interfering nucleogenic isotopes are shown in table S1. All ages were determined relative to the calibration of Carter *et al.* (*39*).

## Supplementary Materials

### The PDF file includes:

Supplementary Text Figs. S1 to S4 Table S1 Legend for data S1 References
