# Supplementary material for: Pliny the Younger advances geochronology
Source: Sci Adv. 2026 Sep 25;12(39):eaeg2624. doi: 10.1126/sciadv.aeg2624 (PMC13618772; doi:10.1126/sciadv.aeg2624)
Supplement: Supplementary file 1 [file sciadv.aeg2624_sm.pdf]

Science Advances
AAAS

Supplementary Materials for

Pliny the Younger advances geochronology

Paul R. Renne *et al.*

Corresponding author: Paul R. Renne, prenne@bgc.org

Sci. Adv. **12**, eaeg2624 (2026)
DOI: 10.1126/sciadv.aeg2624

The PDF file includes:

Supplementary Text
Figs. S1 to S4
Table S1
Legend for data S1
References

Other Supplementary Material for this manuscript includes the following:

Data S1

Age of the eruption from historical records

Pliny's letters

Reconstructions of the date of the Vesuvius eruption typically refer to two letters written by Pliny the Younger to the historian Tacitus. These are known as letters 6.16 and 6.20, the 16th and 20th letters in Book 6 of Pliny's collected correspondence (*22,23*), and are the only surviving eyewitness accounts of the eruption that destroyed Pompeii and Herculaneum.

At the time of the eruption, Pliny the Younger was seventeen years old. His uncle, the famous naturalist and military commander Pliny the Elder, died during a voyage to inspect the eruption more closely, likely overwhelmed by a pyroclastic density current. Pliny the Younger's letters, written almost thirty years later, state the day and month (August 24th), but not the year of the eruption.

Cassius Dio and the year of the eruption

The earliest surviving mention of the year comes from the Roman historian and politician Lucius Cassius Dio, who published his *Roman History (24)* in the early 3rd century CE (ca. 207-219 CE). Cassius Dio's original text does not survive, but is preserved in later paraphrases by the 11th-century historian Xiphilinus and the 12th-century historian Zonaras. In Book 66 of his *Roman History,* Cassius Dio associated the eruption with certain achievements of the emperor Titus: "As a result of these events in Britain, Titus received the title of *imperator* for the fifteenth time. [...] In Campania remarkable and frightful occurrences took place, for a great fire suddenly flared up at the very end of the summer" (*24*). Xiphilinus's Greek is: ἐν μὲν τῇ Βρεττανίᾳ ταῦτ᾽ ἐγένετο, καὶ ἀπ᾽ αὐτῶν ὁ μὲν Τίτος αὐτοκράτωρ τὸ πεντεκαιδέκατον ἐπεκλήθη [...] ἐν δὲ τῇ Καμπανίᾳ φοβερά τινα καὶ θαυμαστὰ συνηνέχθη: πῦρ γὰρ μέγα κατ᾽ αὐτὸ τὸ φθινόπωρον ἐξαπιναίως ἐξήφθη. Zonaras replaces κατ᾽ αὐτὸ τὸ φθινόπωρον with κατὰ τὸ φθινόπωρον ἀθρόον (ἀθρόον = "suddenly").

Dio was writing more than a century after the eruption and did not always follow chronological order. For example, in the same chapter, Dio assigns the circumnavigation of Britain to the same year as "these events in Britain"—but in reality, the circumnavigation happened later, in 83-84 CE (*52*). However, Dio's association of the Vesuvius eruption with the year of Titus's military campaign is generally thought to be reliable. Thus, we can identify the year of the eruption by dating Titus's fifteenth acclamation as *imperator,* an honorific granted to military commanders after a great victory.

Roman chronology: From consuls to the Common Era

In ancient Rome, years were typically identified by significant happenings or by the names of the two consuls—the Roman Republic's highest elected officials, elected annually—who held office at the time. Cassius Dio records that Titus received his fifteenth *imperator* title the same year his father, the emperor Vespasian, died. According to the Roman historian Suetonius's *The Twelve Caesars,* written ca. 121 CE, Vespasian was born "in the consulate of Quintus Sulpicius Camerinus and Gaius Poppaeus Sabinus" (the Latin is: *Vespasianus natus est in Sabinis ultra Reate vico modico, cui nomen est Falacrinae, XV. Kal. Decb. vesperi, Q. Sulpicio Camerino C. Poppaeo Sabino cons., quinquennio ante quam Augustus excederet)* and died "at the age of sixty-nine years, seven months and seven days" (*extinctus est VIIII. Kal. Iul. annum agens aetatis sexagensimum ac nonum superque mensem ac diem septimum*) (*25*). We can therefore

calculate the year of Titus's fifteenth *imperator* title if we know when Quintus Sulpicius Camerinus and Gaius Poppaeus Sabinus were consuls.

The Roman Republic and the later Roman Empire kept detailed records of consuls. Consular dating continued to be used in the Eastern Roman Empire until the mid-sixth century CE, when the emperor Justinian introduced a dating system based on the years of emperors' reigns. The list of Roman consuls spans 1,050 years—from 509 BCE to 541 CE.

The conversion from consular to Common Era dates (BCE/CE) is defined by the sixth-century monk Dionysius Exiguus (*26*). In 525 CE, Dionysius was asked by Pope John I to calculate the upcoming dates of Easter, which was required to fall on the first Sunday after the spring full moon (the first full moon occurring after March 21st). This required consulting astronomical charts compiled by the church of Alexandria. Dionysius was irritated by the fact that the Egyptian church continued to count the years since the accession of the emperor Diocletian (284 CE), whom Dionysius considered an enemy of the church due to his bloody persecution of Christians. To remedy this, Dionysius decided to introduce a new dating system that counted the years *ab incarnatione Domini*—since the birth of Christ. In his completed Easter table, Dionysius stated that the year in which he was writing was 525 *ab incarnatione,* or 525 CE (*53*). Crucially for the conversion from consular to Common Era dates, Dionysius also named one of the acting consuls, Probus Junior. Anchoring Probus Junior to 525 CE, we can convert all consular dates to Common Era dates.

It follows that Q. S. Camerinus and G. P. Sabinus were consuls in 9 CE. Titus's father Vespasian was born that same year and—according to Suetonius's biography—died in June 79 CE. Titus thus became emperor and received his 15th *imperator* title in 79 CE, which, according to Cassius Dio, was the same year Vesuvius erupted.

The conversion of consular to Common Era dates is robust from ~300 BCE onwards, as shown by comparisons with Greek history and Egyptian astronomical tables (*26*). Therefore, the Vesuvius eruption can be confidently assigned to 79 CE.

The day of the eruption

When referring to a specific day of the month, ancient Romans counted forwards to the next of three principal days: the *kalends* (the first day of each month), the *nones* (the 7th day of March, May, July, and October, and the fifth day of every other month), and the *ides* (eight days after the *nones*). The Romans counted days inclusively, meaning they counted both the first and last days of a given interval. For instance, according to inclusive counting, the first and second day of the month are two days apart instead of one.

This system is relevant to Pliny's letters describing the 79 CE Vesuvius eruption. In letter 6.16, Pliny states that the eruption began on the ninth day before the *kalends* of September (*nonum kal. Septembres)*, or August 24th. This is consistent with Cassius Dio's account, which suggests an eruption date at the end of summer. Xiphilinus writes: κατ᾽ αὐτὸ τὸ φθινόπωρον (αὐτὸ = "right at"). Translations from the Greek, here and elsewhere, are from Foss (*22*). Zonaras writes: κατὰ τὸ φθινόπωρον ἀθρόον (ἀθρόον = "suddenly"). Both agree on κατ᾽ τὸ φθινόπωρον, suggesting this is the core of Dio's original phrase (*50*). The word φθινόπωρον ("autumn") is a compound of φθιν(ω) = "decay, wane" and ὀπώρα = "harvest season, late summer". Dio's translators tend to render this as "right at the end of summer". This agrees with the ancient Roman definition of the start of autumn, which was typically placed in early August, e.g. (*27*).

The date debate

Despite Pliny's and Dio's testimony, the August 24th eruption date has been contested. In the late 18th century, Carlo Maria Rosini, a bishop and scholar who had worked on the papyrus scrolls excavated at Herculaneum, doubted the eruption had occurred in August because the excavations had unearthed braziers, carpets, and fruits typically harvested later in the year, including chestnuts, dates, figs, and raisins, e.g. (*28*). Rosini turned to Dio's chapter about the eruption. Because Dio's text had been copied and translated many times in the 1600 years since its publication, various versions were available to Rosini. A 1551 version translated the Greek as *sub Autumni tempus* ("on the eve of autumn"), but included a marginal note suggesting the alternative *autumni extremi tempore* ("at the very end of autumn"). Rosini adopted this alternative. He then proceeded to suggest an eruption date of *non. kal. Decembris,* or November 23rd.

It is important to acknowledge that Rosini's adoption of an "end of autumn" eruption was not based on or supported by the best available manuscripts of Dio's and Pliny's work, and that his proposed date of November 23rd was entirely arbitrary. Nevertheless, the idea that the eruption occurred in the autumn has persisted, enabled by inaccurate transcriptions and translations of Pliny's letters. The oldest known copies of Pliny's correspondence that contain the Vesuvius letters date to the early 9th century and state that the eruption occurred on *nono* (or *nonum) kal. Septembres* (August 24th). However, in later manuscripts, *nonum* was sometimes misspelled as *novum*. Because the phrase *novum kal. Septembres* is nonsensical, it was "corrected" by later editors, who changed it to *nou* and then to *novemb.* This error was included in a printed edition in the late 15th century, which was widely circulated and is still occasionally cited as evidence that the eruption occurred in late autumn. However, that is not what Pliny wrote. The oldest, most reliable manuscripts of Pliny's letters read *non. kal. Septembres,* cementing August 24th as the most likely date of the eruption. For more information about the manuscript tradition of Pliny's letters, interested readers are referred to Pedar W. Foss's *Pliny and the Eruption of Vesuvius* (*22*).

Archeological finds are often used to support a late autumn eruption date and they tend to attract considerable media attention. For example, in 2018, a charcoal graffito beginning *xvi k nov* (October 17th) was found on a wall in Pompeii (*29*). The graffito has been used to argue that Vesuvius could not have erupted before October because charcoal writing is too fragile to survive many months in the open. However, an experiment conducted recently in Pompeii showed that charcoal graffiti can easily last from October 17th until August 24th the following year (*30*). As for the presence of braziers, autumn fruits, and warm clothing worn by victims of the eruption: Braziers can be used for cooking, not just for heating in cold weather; people in Pompeii and Herculaneum may well have donned heavy woolen clothing to protect themselves from the scorching eruption fallout (Pliny the Younger described people tying pillows to their heads for the same purpose); and dried fruits such as raisins, dates, or chestnuts found at Pompeii could be consistent with an end-August eruption. In any case, dried fruits should not be used to date the eruption because they can be stored for many months and could have been harvested in previous years (*27*).

Proponents of a late autumn eruption often refer to a coin hoard found in Pompeii's "House of the Golden Bracelet". One of the coins celebrates Titus's fifteenth *imperator* title—the same event Cassius Dio used to date the eruption. However, Dio did not specify whether the eruption occurred before or after Titus's title, only that both events occurred in the same year.

The coin from Pompeii is corroded and the inscriptions are not easily legible, but by comparing it to similar coins in better condition, D'Ambrosio *et al.* (*31*) inferred that its reverse inscription reads: *IMP TITUS CAES VESPASIAN AVG PM / TR P VIIII IMP XV COS VII PP* (*Imperator Titus Caesar Vespasianus Augustus pontifex maximus, tribunicia potestate VIIII, imperator XV, consul VII, pater patriae)*. For the purpose of dating the Vesuvius eruption, the relevant part is *IMP XV*, which implies the coin was minted after Titus received the title of *imperator* for the fifteenth time.

Titus's fifteenth *imperator* title can be dated using historical documents. A bronze copy of a letter from Titus to the Spanish city of Munigua is signed *IMP XIIII* and dated September 7th (*54*). A bronze military diploma from Egypt bears the same signature and is dated *ad vi idus sept* (September 8th) (*55*). We know these documents date to 79 CE because Titus signs himself *P P* (*pater patriae* = "father of the fatherland"), a title borne by Roman emperors; thus, when the documents were written, Titus had already succeeded his father after the latter's death in June 79 CE. The two documents show that Titus was still signing himself *IMP XIIII* until at least September 8, 79 CE. The coin found in Pompeii apparently reads *IMP XV* and so, the argument goes, it must have been minted after that date.

However, the original catalog by D'Ambrosio et al. (*31*) did not include an image of the Pompeii coin. Abdy (*32*) reexamined the coin and found that it had been misinterpreted. Comparing it to coins in better condition, he concluded that the Pompeii coin was more similar to a mint reading *IMP XIIII* rather than *IMP XV*. This indicates Titus was still *IMP XIIII* when the Pompeii coin was minted—perhaps earlier than September 8th. Importantly, if the coin reads *IMP XIIII,* it can neither confirm nor refute an August eruption date.

Abdy (*32*) notes that, based on Titus's political career, three generations of coins are candidate analogs for the Pompeii coin. Titus did not become emperor until after his father's death on June 23rd, 79 CE, and he did not receive his fifteenth *imperator* title until autumn, so coins minted with his image before June 23rd should name him *TR P VIIII* ***IMP XIIII*** *COS VII* (*tribunicia potestate VIIII, imperator XIIII, consul VII).* When Titus became emperor, he gained the title *pater patriae*, so coins minted after that date should read *TR P VIIII* ***IMP XIIII*** *COS VII* ***P P***. Sometime after September 14th, Titus received his fifteenth *imperator* title and coins minted after that read *TR P VIIII* ***IMP XV*** *COS VII* ***P P.***

Crucially, there is no sign of the initials *P P* on the Pompeii coin, suggesting that it was minted before Titus became emperor in June 79 CE. This and other important features are highlighted in Fig. S1.

<u>Concluding remarks</u>

Although later reinterpretations have proposed autumn or even November dates, there is no solid reason to doubt Pliny's and Cassius Dio's accounts. The best available evidence points to August 24th, 79 CE as the date of the Vesuvius eruption, and that is the date we use as a reference point.

Previous $^{40}Ar/^{39}Ar$ Dating

Renne et al. (*15*) analyzed samples from pumice collected at the Villa Poppea in Oplontis. The pumice was described by Renne et al. (*15*), who did not collect the sample, as coming from the lower white pumice eruptive unit. However, that pumice is distinctly darker in

color than the pumice sample used in the present study, which was collected by Andrea Marzoli. We suspect that the previous sample was actually taken from the upper grey eruptive unit (EU3) described by Cioni et al. (*51*). Renne et al. (*15*) performed laser incremental heating $^{40}Ar/^{39}Ar$ analysis of 12 multigrain aliquots of sanidine and reported an inverse isochron age of 1925 ± 94 years (trapped $^{40}Ar/^{36}Ar$ = 306.9 ± 1.5, MSWD = 0.51) for all 46 steps. These results were based on a neutron irradiation of 3 min duration and an age of 1.194 Ma for the Alder Creek sanidine (ACs) standard, an atmospheric $^{40}Ar/^{36}Ar$ of 295.5, and a value of (8 ± 3) x 10^{-4} for reactor-produced $^{40}Ar/^{39}Ar$ derived from K. The reported age in calendar years is 72 CE ±94 CE. Renne and Min (*56*) reported additional isochron ages of 1889 ±132 and 1936 ±207 years (uncorrected for time since publication) but these results were fraught with analytical problems and are not considered reliable enough to warrant further discussion.

Lanphere et al. (*16*) analyzed samples from pumice in unspecified eruptive units of the 79 CE deposits in Pompei and Oplontis. They used resistance furnace incremental heating $^{40}Ar/^{39}Ar$ analysis of 8 multigrain aliquots of sanidine. to obtain plateau ages whose weighted mean was reported as 1921 ± 66 years (in 2000 CE). These results were based on a neutron irradiation of 30 min duration and an age of 28.92 Ma for the Taylor Creek sanidine (TCs) standard. This age must be a typographical error, as in other USGS publications of this vintage the age of TCs is reported as 27.92 Ma; we use this latter value to recalculate the Lanphere *et al*. (2007) results. Lanphere *et al*. (2007) reported a value of 0.0000 ± 0.0000 for reactor-produced $^{40}Ar/^{39}Ar$ derived from K. The reported age in calendar years is 79 CE ± 66 CE.

There are some important differences between the parameters used in these two studies; these differences must be accounted for in order to compare the results. Although different standards were used in the two studies, the results can be normalized to a common standard (Fish Canyon sanidine; FCs) using methods outlined by Reiners et al. (*57*) equations 9.30-9.32b and using the intercalibration factors between FCs and ACs of Niespolo et al. (*49*) and between FCs and TCs of Fleck et al. (*58*) to normalize the results of (15) and (16), respectively. This procedure produces *R*-values of Vesuvius relative to FCs of 6.8149 x 10^{-5} (15) and 6.9769 x 10^{-5} (16), corresponding to calendar ages of 61 CE± 94 CE and 125 CE ± 66 CE, respectively, using the calibration of Carter et al. (*39*). Within the relatively large uncertainties, both are indistinguishable from the canonical age of 79 CE.

The second difference between the two studies is the correction for reactor-produced $^{40}Ar/^{39}Ar$ derived from K ($(^{40}Ar/^{39}Ar)_K$). Lanphere et al. (16) reported using a remarkably precise value of 0.0000 ±0.0000 without any documentation, which was ascribed to the use of Cd shielding to reduce the thermal neutron fluence. This is difficult to reconcile with the 7 values for various reactors summarized by McDougall and Harrison (*59*), wherein all the values reported for irradiations with Cd shielding are >1 x 10^{-4}. Moreover, using the same reactor (Geological Survey Triga Reactor; GSTR) as Lanphere et al. (16), Fleck et al. (58) reported data from 9 Cd-shielded irradiations whose weighted mean $(^{40}Ar/^{39}Ar)_K$ is (1.04 ±1.00) x 10^{-3}. Thus we find that a value of 0.0000 ±0.0000 lacks credibility.

Importance of the potassium correction

Lanphere et al. (16) did not report *J*-values for their samples, hence the neutron fluence in their irradiations is not directly known as the reactor power was not specified. However, GSTR records indicate that the two irradiations in question were conducted at 1MW, corresponding to *J*/hour of 2.5×10^{-2}/hour (*60*). For a 0.5 hour irradiation, this translates to spurious age excesses of 23 to 227 years for values of $(^{40}Ar/^{39}Ar)_K$ between 10^{-4} and 10^{-3}, respectively (Figure S1). Assuming that the $(^{40}Ar/^{39}Ar)_K = (4.54 \pm 0.26) \times 10^{-4}$ in the GSTR reactor is the same as the OSTR (see section below), and neglecting $(^{36}Ar/^{39}Ar)_K$, the excess age would be 102 years. Thus, the updated and corrected calendar age of Lanphere et al. (*16*) would be 23 CE. This is probably still indistinguishable from the calendar age of 79 CE when all sources of uncertainty are considered. Analogously, the updated calendar age of Renne et al. (*15*) is 73 CE when corrected to the same value of $(^{40}Ar/^{39}Ar)_K$. We assume that the delay between irradiation and analysis was brief for Lanphere et al. (*16*), which we can verify for Renne et al. (*15*), hence the ingrowth of $^{36}Ar_K$ was insignificant.

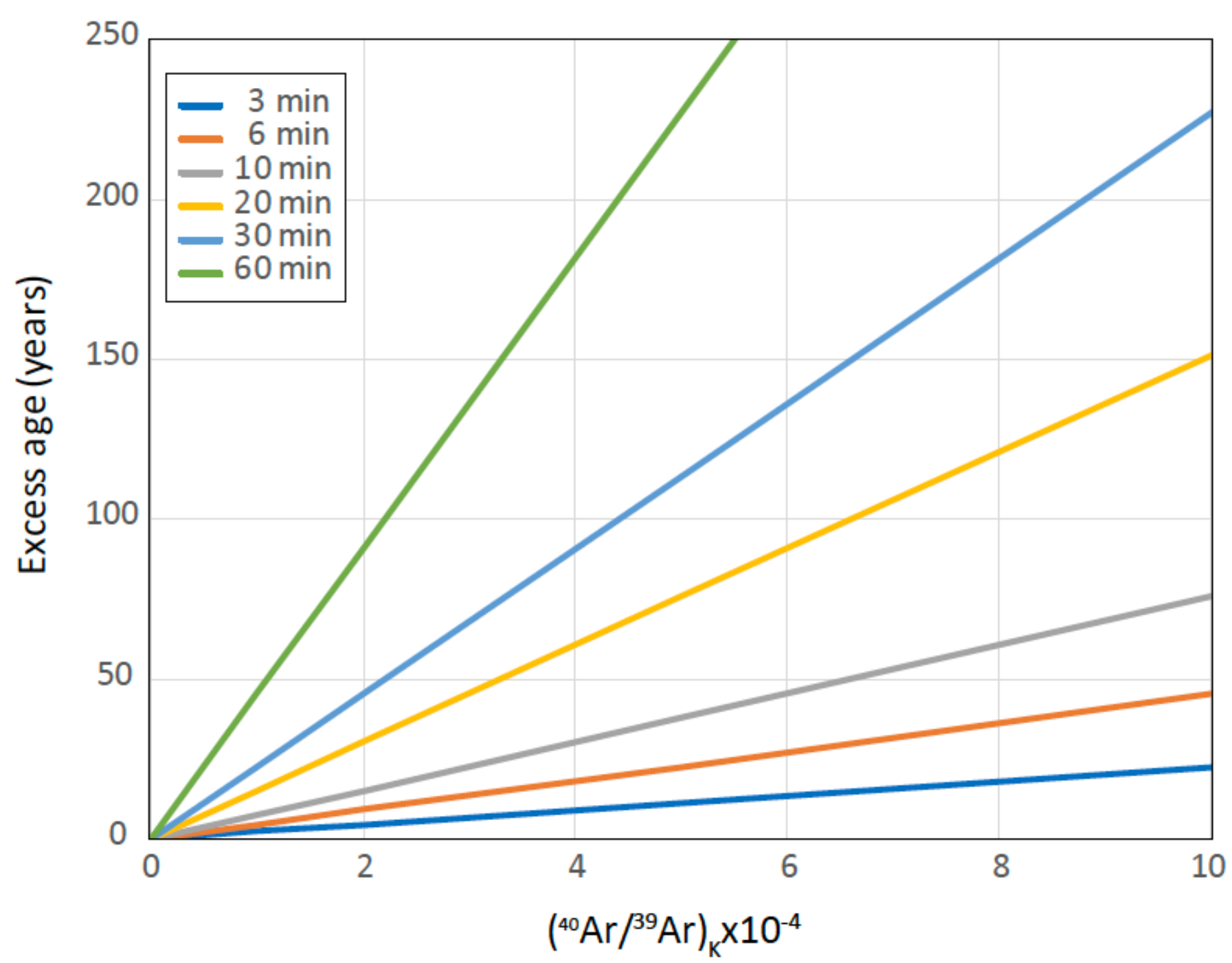

Fig. S1. Excess (spurious) age as a function of $(^{40}Ar/^{39}Ar)_K$ for various irradiation times from 3 to 60 minutes duration. Specific values based on data for the OSTR reactor (see text).

As shown above, knowledge of the $(^{40}Ar/^{39}Ar)_K$ is critical to accurate and precise $^{40}Ar/^{39}Ar$ dating on the millennial timescale. The customary method for determining this value is to irradiate high K material lacking elements with Z greater than that of potassium so that all Ar isotopes produced are derived from K via the reaction $^{39}K(n, p)^{39}Ar$ and to a lesser extent $^{40}K(n, d)^{39}Ar$. Accurate determination of this value is challenging due to the small amount of ^{40}Ar produced in the reactor compared with the inevitable contamination from atmospheric ^{40}Ar. The correction for atmospheric ^{40}Ar is complicated by the fact that ^{36}Ar is also produced from K in the reactor by the $^{39}K(n, \alpha)^{36}Cl$ reaction followed by β^- decay of ^{36}Cl to ^{36}Ar (*38*). Due to the long (3.01×10^5 years) half-life of ^{36}Cl, ^{36}Ar

ingrowth is generally negligible if analysis occurs within a few months after neutron irradiation (*38*), but the effect is largest for very young samples that are proportionately most affected by $(^{40}Ar/^{39}Ar)_K$. Further, as discussed below, ppm level concentrations of Ca in K-bearing minerals contribute ^{36}Ar that must quantified and corrected. To our knowledge, none of the previous determinations of $(^{40}Ar/^{39}Ar)_K$ have taken $^{36}Ar_K$ into account.

The value of $(^{40}Ar/^{39}Ar)_K$ is determined as:

$$\left(\frac{^{40}Ar}{^{39}Ar}\right)_K = \frac{(^{40}Ar_T - {}^{40}Ar_A)}{(^{39}Ar_T - {}^{39}Ar_{Ca})} \tag{1}$$

where $^{40}Ar_A = {}^{36}Ar_A \cdot 298.56$ (2)

and $^{39}Ar_{Ca} = {}^{37}Ar_T \cdot \left(\frac{^{39}Ar}{^{37}Ar}\right)_{Ca}$ (3)

and $^{36}Ar_A = {}^{36}Ar_T - {}^{36}Ar_K - {}^{36}Ar_{Ca}$ (4)

and $^{36}Ar_K = {}^{39}Ar_T \cdot \left(\frac{^{36}Ar}{^{39}Ar}\right)_K$ (5)

and $^{36}Ar_{Ca} = {}^{37}Ar_T \cdot \left(\frac{^{36}Ar}{^{37}Ar}\right)_{Ca}$ (6)

and $\left(\frac{^{36}Ar}{^{39}Ar}\right)_K = \left(\frac{^{36}Cl}{^{39}Ar}\right)_K \frac{(1-e^{-\lambda_{36}\cdot\Delta t})}{(e^{-\lambda_{39}\cdot\Delta t})}$ (7)

Subscripts in the above indicate sources: atmospheric (*A*), potassium (*K*), and total measured (*T*) values corrected for background, mass discrimination, detector gain variations, and decay. λ_{36} and λ_{39} are the decay constants of ^{36}Cl and ^{39}K, respectively.

The above equations assume ^{37}Ar is produced entirely from Ca, which deviates from the general assumption that ^{37}Ar is also produced from K. Correction factors for ^{37}Ar production from K have traditionally been determined in experiments on high K material assumed to be devoid of Ca. However, the reaction threshold for the production of ^{37}Ar from K is ca. 10 MeV and therefore production should be dominated by Ca at ppm level concentrations in high K materials. For example, ORIGEN simulations of the GODIVA critical assembly (fission spectrum neutrons) yield flux-averaged cross sections for $^{40}Ca(n,a)^{37}Ar$ and $^{39}K(n,n+d)^{37}Ar$ of 2.27×10^{-2} and 2.48×10^{-8} barns, respectively (*61*). Thus, the crossover at which production from Ca exceeds that from K would be ca. 1 ppm Ca in pure K, or a fraction of a ppm in K-bearing silicates. Even trace-metal grade reagents contain 10s of ppm contaminants, and it is unlikely such reagents were feedstock in the manufacturing of the K- glass (nominally Fe-doped $KAlSiO_4$ composition) utilized to determine reactor interference corrections. Further, on a plot of $^{38}Ar/^{39}Ar$ vs. $^{37}Ar/^{39}Ar$ obtained from K-glass data (Table S1), a linear correlation with a non-zero y-intercept is observed, which suggests there are two sources of ^{38}Ar, one of which is more closely associated with ^{39}Ar and one of which is more closely associated with ^{37}Ar. In this case, the intercept likely reflects $(^{38}Ar/^{39}Ar)_K$ and the second component is a mixture of $^{39}Ar_{Ca}$, $^{38}Ar_{Ca}$, and $^{37}Ar_{Ca}$ (with atmospheric ^{38}Ar contributing scatter). It is therefore likely that the production of ^{37}Ar from K can be neglected in geochronology.

Strictly speaking, the value of $(^{36}Cl/^{39}Ar)_K$ in equation (5) is specific to a given neutron energy spectrum, hence may vary between reactors or even at different locations within a reactor, and whether or not any shielding is used. Carter et al. (*38*) estimated this value to be 0.4 ± 0.01 for the Cadmium-lined in-core irradiation tube (CLICIT) facility at the Oregon State University Triga reactor. We determined this value independently along with the $(^{40}Ar/^{39}Ar)_K$ as follows.

Determination of $(^{40}Ar/^{39}Ar)_K$ and $(^{36}Cl/^{39}Ar)_K$

We determined the relative production rates of ^{36}Cl with respect to ^{39}Ar from K $(^{36}Cl/^{39}Ar)_K$ and ^{40}Ar with respect to ^{39}Ar from K $(^{40}Ar/^{39}Ar)_K$ by analyzing "zero-age" K-rich glass from two irradiations, one from 2009 (100 hours) and one from 2025 (40 hours). Complete analytical data are provided in Table S1.The K-glass has the composition of Fe-doped kalsilite ($KAlSiO_4$). It was manufactured in ca. 1990 by Corning Inc. in air and subsequently degassed under UHV conditions in a resistance furnace to reduce atmospheric Ar contamination. The radiogenic ingrowth $^{40}Ar^*/^{39}Ar_K$ ratio is <0.2% of the nucleogenic production ratio and was therefore neglected in our calculation. To determine $(^{36}Cl/^{39}Ar)_K$ and $(^{40}Ar/^{39}Ar)_K$, we used three isotope plots of $^{36}Ar/^{40}Ar$ vs. $^{39}Ar/^{40}Ar$, where the slope (m) is proportional to the production ratios according to the following:

$$m = \left(\frac{^{36}Cl}{^{39}Ar}\right)_K \left(1 - e^{-\lambda_{Cl} t}\right)\mathcal{B} - \left(\frac{^{40}Ar}{^{39}Ar}\right)_K b\,, \qquad (8)$$

where $\mathcal{B}$ is the branching fraction (98.1%) for ^{36}Cl β^- decay to ^{36}Ar, λ_{Cl} is the ^{36}Cl decay constant (3.013 ±0.015) x10^5 y^{-1}; (*62*)), t is the time elapsed since the end of irradiation, and b is the y-intercept, assumed here to be the atmospheric $^{36}Ar/^{40}Ar$ ratio. All data were corrected for contributions from Ca as described above. The two irradiations yield distinct slopes, from which $(^{36}Cl/^{39}Ar)_K$ can be determined according to the following:

$$\left(\frac{^{36}Cl}{^{39}Ar}\right)_K = \frac{1}{\mathcal{B}}\left(\frac{m_1 - m_2}{e^{-\lambda_{Cl} t_2} - e^{-\lambda_{Cl} t_1}\right). \qquad (9)$$

One can then determine $(^{40}Ar/^{39}Ar)_K$ by solving Eq. (1) using either of the two slopes and the associated time elapsed since the end of irradiation. For both regressions, we fixed the y-intercept (b) to the isotopic composition of atmospheric Ar ($^{36}Ar/^{40}Ar$ = 0.003349; (*35*). We neglected uncertainties in $\mathcal{B}$ and λ_{Cl}. Data are shown in Figure S2A and S2B. The inferred $(^{36}Cl/^{39}Ar)_K$ and $(^{40}Ar/^{39}Ar)_K$ ratios are (2.835 ±0.025) x 10^{-1} and (4.54 ±0.26) x 10^{-4}, respectively.

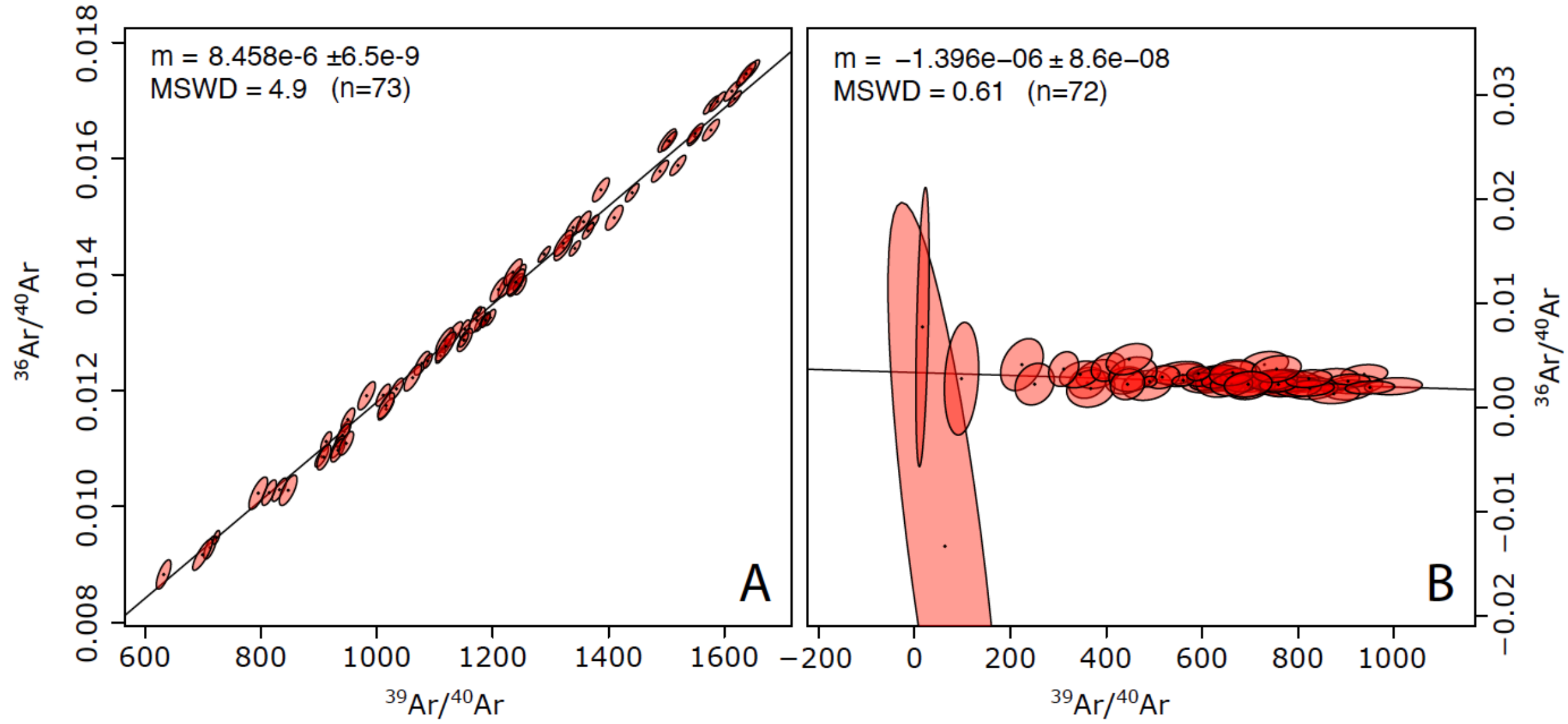

Fig. S2. Three isotope plots of data obtained from the analysis of "zero-age" K-glass. Ellipses denote 1σ uncertainties, with rotation reflecting uncertainty correlations. The data were regressed with and the figures were modified from IsoplotR (*34*). The left panel (A) shows data from samples that were irradiated in 2009, which allowed for significant ingrowth of ^{36}Ar from ^{36}Cl. The right panel (B) shows data from samples that were irradiated in 2025. Both datasets were generated in 2025.

Determination of full posterior of $\lambda_{EC,\beta+}$

We used an affine-invariant Markov Chain Monte Carlo (MCMC) sampler (*50*) to jointly estimate the ^{40}K decay constants and calibration parameters needed to place the Vesuvius measurements on an absolute timescale. Sampled parameters include the eruption age (t_{AM1}), the K-Ar isotopic composition of Fish Canyon sanidine (FCs) $(^{40}Ar^{*}/^{40}K)_{FCs}$, and the *R*-value relating AM1 to FCs, R_{FCs}^{AM1}. Sampling these quantities together allows uncertainties in measurements and priors, as well as parameter covariances, to be handled consistently through the model (Figure S4). This joint posterior sampling yields a tightly constrained estimate of $\lambda_{EC,\beta+}$, which is the key result of this analysis (Table S1). Posterior summaries are reported in Table S1 and illustrated in Figure S3.

Chains were initialized near the centers of their prior distributions and run with 64 parallel walkers/chain for 10^5 steps. The first 5 x 10^4 steps were discarded as burn-in and the remaining chains were thinned to reduce autocorrelation effects. Convergence was assessed using integrated autocorrelation times estimated from the chains and by verifying that the total chain length exceeded at least 50 times the largest autocorrelation time across parameters. Mean acceptance rates were additionally monitored (~59% of proposed samples were accepted) to ensure efficient sampling. Together, these diagnostics indicated adequate mixing and effective sampling of the posterior.

The resulting posterior yields a tightly constrained estimate of $\lambda_{EC,\beta+}$ (Table S1; Figure S3). This value is statistically indistinguishable from the approximation presented in the main text, demonstrating that the simpler approach and complete Bayesian treatment converge on the same result.

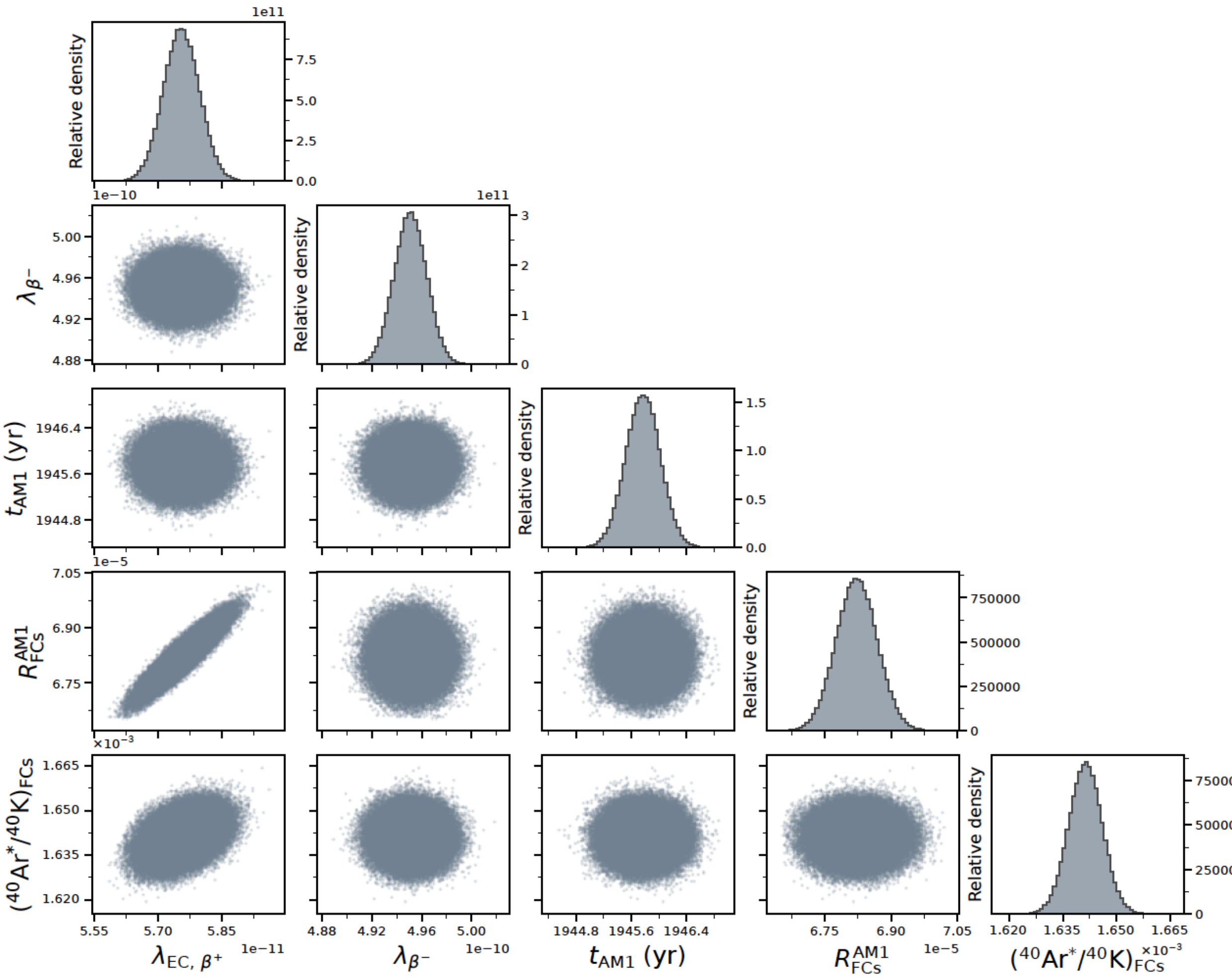

Fig. S3. Corner plot showing the joint posterior distributions of the ^{40}K decay constants, ^{40}Ar*/^{40}K of FCs and R_{FCs}^{AM-1}. The diagonal panels show the marginal posterior for each parameter (normalized histograms), and off-diagonal lower left panels show pairwise relationships using plots of the retained MCMC samples. Elongated or tilted point clouds indicated posterior correlations among parameters – for example both $\lambda_{EC,\beta+}$ and R_{FCs}^{AM-1}.

Table S1. Summary of MCMC results

Parameter	Prior	Posterior (68% credible level)
$\lambda_{EC,\beta+}$	U(0.0, 6×10^{-10}, 5.9×10^{-10}) a^{-1} [a]	(5.7550 ± 0.042)×10^{-10} a^{-1}
$\lambda_{\beta-}$	(4.9510 ± 0.0131) ×10^{-9} a^{-1} [b]	(4.9510 ± 0.0131) ×10^{-9} a^{-1}
R_{FCs}^{AM1}	(6.8223 ± 0.0460) ×10^{-5} [c]	(6.8225 ± 0.046) ×10^{-5}
(^{40}Ar*/^{40}K)$_{FCs}$	(1.6413 ±0.0047) × 10^{-3} [c]	(1.6413 ±0.0047) × 10^{-3}
t_{AM1}	1945.77 ± 0.25 a[d]	1945.77 ± 0.25 a

[a] Denotes uniform (U) distribution bounded between lower and upper values stated in the brackets.
[b] Prior is taken from Kossert et al. (*27*)
[c] Derivation of prior is given in the main text.
[d] AM1 prior is constructed from time of analysis to the 24th August eruption date and with 3 month uncertainty.

Sample-Standard Irradiation Geometry

Accurate and precise determination of the neutron fluence parameter *J* corresponding to each sample's position in the reactor is an important factor in the age uncertainty budget. In this study we used the weighted average of analyses from each well containing standards (ACs) to assign a *J*-value to that well. *J*-values for each well containing AM-1 were then determined as the weighted mean of J-values determined from the three wells most closely bracketing it.

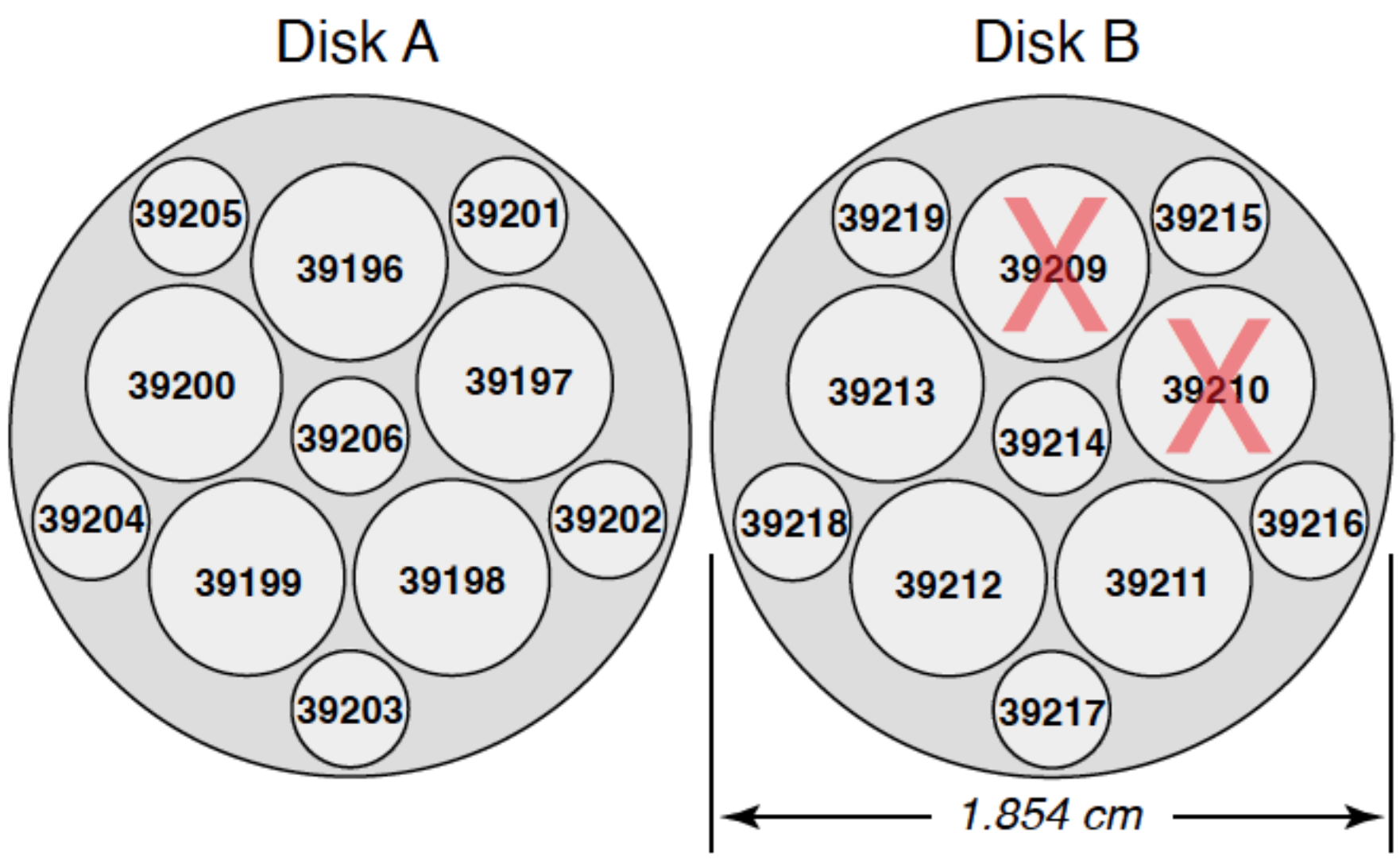

Fig. S4. Irradiation disks showing spatial relations between standards (ACs) and unknowns (AM-1) during neutron irradiation. Small circles represent wells 2.87 mm diameter x 2.03 mm deep, containing ACs. Large circles represent wells 5.33 mm diameter x 3.30 mm deep, containing AM-1. Large wells of AM-1 with X (#s 39209 and 39210) are suspect of contamination and all analyses of material from these wells are excluded from the age determination.

Data S1. (separate file) Argon isotope data

REFERENCES

1. D. J. Lowe, Tephrochronology and its application: A review. *Quat. Geochronol.* **6**, 107–153 (2011).

2. M. D. Schmitz, B. S. Singer, A. D. Rooney, in *Geologic Time Scale 2020*, F. M. Gradstein, J. G. Ogg, M. D. Schmitz, G. M. Ogg, Eds. (Elsevier, 2020), pp. 193–209.

3. G. Mannella, B. Giaccio, G. Zanchetta, E. Regattieri, E. M. Niespolo, A. Pereira, P. R. Renne, S. Nomade, N. Leicher, N. Perchiazzi, B. Wagner, Palaeoenvironmental and palaeohydrological variability of mountain areas in the central Mediterranean region: A 190 ka-long chronicle from the independently dated Fucino palaeolake record (central Italy). *Quat. Sci. Rev.* **210**, 190–210 (2019).

4. V. Ponomareva, M. Portnyagin, S. M. Davies, Tephra without borders: Far-reaching clues into past explosive eruptions. *Front. Earth Sci.* **3**, 83 (2015).

5. P. R. Renne, A. L. Deino, F. J. Hilgen, K. F. Kuiper, D. F. Mark, W. S. Mitchell III, L. E. Morgan, R. Mundil, J. Smit, Time scales of critical events around the Cretaceous-Paleogene boundary. *Science* **339**, 684–687 (2013)

6. J. Martí Molist, in *Oxford Handbook Topics in Physical Sciences*, Oxford Handbooks Editorial Board (Oxford Univ. Press, 2017).

7. W. Marzocchi, M. S. Bebbington, Probabilistic eruption forecasting at short and long time scales. *Bull. Volcanol.* **74**, 1777–1805 (2012).

8. P. G. Albert, B. Giaccio, R. Isaia, A. Costa, E. M. Niespolo, S. Nomade, A. Pereira, P. R. Renne, A Hinchliffe, D. F. Mark, R. J. Brown, V. C. Smith, Evidence for a large-magnitude eruption from Campi Flegrei caldera (Italy) at 29 ka. *Geology* **47**, 595–599 (2019).

9. B. Giaccio, I. Hajdas, R. Isaia, A. Deino, S. Nomade, High-precision ^{14}C and $^{40}Ar/^{39}Ar$ dating of the Campanian Ignimbrite (Y-5) reconciles the time-scales of climatic-cultural processes at 40 ka. *Sci. Rep.* **7**, 45940 (2017).

10. A. L. Deino, G. Orsi, S. de Vita, M. Piochi, The age of the Neapolitan Yellow Tuff caldera-forming eruption (Campi Flegrei caldera Italy) assessed by Ar-40/Ar-39 dating method. *J. Volcanol. Geotherm. Res.* **133**, 157–170 (2004).

11. P. Tierz, Long-term probabilistic volcanic hazard assessment using open and non-open data: Observations and current issues. *Front. Earth Sci.* **8**, 257 (2020).

12. J. J. Bösken, C. Schmidt, Direct and indirect luminescence dating of tephra: A review. *J. Quat. Sci.* **35**, 39–53 (2020).

13. J. G. Ogg, in *Geologic Time Scale 2020*, F. M. Gradstein, J. G. Ogg, M. D. Schmitz, G. M. Ogg, Eds. (Elsevier, 2020), pp. 159–192.

14. K. Preece, D. F. Mark, J. Barclay, B. E. Cohen, K. J. Chamberlain, C. Jowitt, C. Vye-Brown, R. J. Brown, S. Hamilton, Bridging the gap: $^{40}Ar/^{39}Ar$ dating of volcanic eruptions from the 'Age of Discovery'. *Geology* **46**, 1035–1038 (2018).

15. P. R. Renne, W. D. Sharp, A. L. Deino, G. Orsi, L. Civetta, $^{40}Ar/^{39}Ar$ dating into the historical realm: Calibration against Pliny the Younger. *Science* **277**, 1279–1280 (1997).

16. M. Lanphere D. Champion, L. Melluso, V. Morra, A. Perrota, C. Scarpati, D. Tedesco, A. Calvert, $^{40}Ar/^{39}Ar$ ages of the AD 79 eruption of Vesuvius, Italy. *Bull. Volcanol.* **69**, 259–263 (2007).

17. D. M. Doronzo, M. A. Di Vito, I. Arienzo, M. Bini, B. Calusi, M. Cerminara, S. Corradini, S. de Vita, B. Giaccio, L. Gurioli, G. Mannella, G. P. Ricciardi, I. Rucco, D. Sparice, M. Todesco, E. Trasatti, G. Zanchetta, The 79 CE eruption of Vesuvius: A lesson from the past and the need of a multidisciplinary approach for developments in volcanology. *Earth Sci. Rev.* **231**, 104072 (2022).

18. S. Aciego, B. M. Kennedy, D. J. DePaolo, J. N. Christensen, I. Hutcheon, U-Th/He age of phenocrystic garnet from the 79 AD eruption of Mt, Vesuvius. *Earth Planet. Sci. Lett.* **216**, 209–219 (2003).

19. R. Sacchi, A. Cutignano, G. Picariello, A. Paduano, A. Genovese, F. Siano, G. Nuzzo, S. Caira, C. Lubritto, P. Ricci, A. D'Auria, G. di Pasquale, A. Motta, F. Addeo, Olive oil from the 79 AD Vesuvius eruption stored at the Naples National Archaeological Museum (Italy). *Npj Sci. Food* **4**, 19 (2020).

20. C. Barbante, N. M. Kehrwald, P. Marianelli, B. M. Vinther, J. P. Steffensen, G. Cozzi, C. U. Hammer, H. B. Clausen, M.-L. Siggaard-Andersen, Greenland ice core evidence of the 79 AD Vesuvius eruption. *Clim. Past* **9**, 1221–1232 (2013).

21. C. G. Newhall, S. Self, The volcanic explosivity index (VEI) an estimate of explosive magnitude for historical volcanism. *J. Geophys. Res. Oceans* **87**, 1231–1238 (1982).

22. P. W. Foss, *Pliny and the Eruption of Vesuvius* (Routledge, 2022).

23. Pliny the Younger, "Letters" in *Letters and Panegyricus*, trans. B. Radice (Harvard Univ. Press, 1969).

24. L. C. Dio, *Dio's Roman History*, vol. 8, trans. E. Cary (Heinemann, 1925).

25. G. S. Tranquillus, *Lives of the Caesars*, vol. 2, trans. J. C. Rolfe (Heinemann, 1914).

26. E. J. Bickerman, *Chronology of the Ancient World* (Cornell Univ. Press, ed. 3, 1980).

27. M. T. Varro, "On Agriculture" in *Cato and Varro on Agriculture*, trans. W. D. Hooper, H. B. Ash (Harvard Univ. Press, 1934).

28. G. Rolandi, A. Paone, M. Di Lascio, G. Stefani, The 79 AD eruption of Somma: The relationship between the date of the eruption and the southeast tephra dispersion. *J. Volcanol. Geotherm. Res.* **169**, 87–98 (2008).

29. M. Osanna, *Pompei. Il Tempo Ritrovato: Le Nuove Scoperte* (Rizzoli, 2019).

30. G. Zuchtriegel, C. Comegna, F. Conte, A. Russo, La data della distruzione di Pompei: Premesse per un dibattito aperto. *E-Journal Scavi Di Pompei* **27**, 2–25 (2024).

31. A. D'Ambrosio, P. G. Guzzo, M. Mastroroberto, *Storie da un'Eruzione: Pompei, Ercolano, Oplontis* (Electa, 2003).

32. R. Abdy, The last coin in pompeii: A re-evaluation of the coin hoard from the house of the golden bracelet. *Numis. Chron.* **173**, 79–83 (2013).

33. I. Wendt, C. Carl, The statistical distribution of the mean squared weighted deviation. *Chem. Geol.* **86**, 275–285 (1991).

34. P. Vermeesch, IsoplotR: A free and open toolbox for geochronology. *Geosci. Front.* **9**, 1479–1493 (2018).

35. J. Y. Lee, K. Marti, J. P. Severinghaus, K. Kawamura, H. S. Yoo, J. B. Lee, J. S. Kim, A redetermination of the isotopic abundances of atmospheric Ar. *Geochim. Cosmochim. Acta* **70**, 4507–4512 (2006).

36. P. R. Renne, Implications of sanidine K/Ca compositions for $^{40}Ar/^{39}Ar$ geochronology. *Geochim. Cosmochim. Acta* **407**, 91–104 (2025).

37. S. E. Cox, S. R. Hemming, D. Tootell, The Isotopx NGX and ATONA Faraday amplifiers. *Geochronology* **2**, 231–243 (2020).

38. J. N. Carter, P. R. Renne, L. E. Morgan, Potassium-39-derived ^{36}Ar production during fission-neutron irradiation and its effect on $^{40}Ar/^{39}Ar$ ages. *Geochim. Cosmochim. Acta* **357**, 26–34, (2023).

39. J. N. Carter, C. E. J. Hasler, A. J. Fuentes, A. J. Tholt, L. E. Morgan, P. R. Renne, Bayesian calibration of the ^{40}K decay scheme with implications for ^{40}K-based geochronology. *Geochim. Cosmochim. Acta* **397**, 149–163 (2025).

40. P. J. Reimer, W. E. N. Austin, E. Bard, A. Bayliss, P. G. Blackwell, C. Bronk Ramsey, M. Butzin, H. Cheng, R. L. Edwards, M. Friedrich, P. M. Grootes, T. P. Guilderson, I. Hajdas, T. J. Heaton, A. G. Hogg, K. A. Hughen, B. Kromer, S. W. Manning, R. Muscheler, J. G. Palmer, C. Pearson, J. van der Plicht, R. W. Reimer, D. A. Richards, E. M. Scott, J. R. Southon, C. S. M.

Turney, L. Wacker, F. Adolphi, U. Büntgen, M. Capano, S. M. Fahrni, A. Fogtmann-Schulz, R. Friedrich, P. Köhler, S. Kudsk, F. Miyake, J. Olsen, F. Reinig, M. Sakamoto, A. Sookdeo, S. Talamo, The INTCAL20 Northern hemisphere radiocarbon age calibration curve (0-55 CAL KBP). *Radiocarbon* **62**, 725–757 (2020).

41. L. Yang, F. Wang, H. Feng, L. Wu, W. Shi, $^{40}Ar/^{39}Ar$ geochronology of Holocene volcanic activity at Changbaishan Tianchi volcano, Northeast China. *Quat. Geochronol.* **21**, 106–114 (2014).

42. C. Oppenheimer, L. Wachker, J. Xu, J. D. Galván, M. Stoffel, S. Guillet, C. Corona, M. Sigl, N. Di Cosmo, I. Hajdas, B. Pan, R. Breuker, L. Schneider, J. Esper, J. Fei, J. O. S. Hammond, U. Büntgen, Multi-proxy dating the 'Millenium Eruption' of Changbaishan to late 946 CE. *Quat. Sci. Rev.* **158**, 164–171 (2017).

43. F. Ramos, M. T. Heizler, J. E. Buettner, J. B. Gill, H. Q. Wei, C. A. Dimond, S. R. Scott, U-series and $^{40}Ar/^{39}Ar$ ages of Holocene volcanic rocks at Changbaishan volcano, China. *Geology* **44**, 511–514 (2016).

44. K. Kossert, Y. Amelin, D. Arnold, R. Merle, X. Mougeot, M. Schmiedel, D. Zapata-Garcia, Activity standardization of two enriched K-40 solutions for the determination of decay scheme parameters and the half-life. *Appl. Radiat. Isot.* **188**, 110362 (2022).

45. A. H. Jaffey, K. F. Flynn, L. E. Glendenin, W. C. Bentley, A. M. Essling, Precision measurement of half-lives and specific activities of U-235 AND U-238. *Phys. Rev. C* **4**, 1889–1906 (1971).

46. R. Schon, G. Winkler, W. Kutschera, A critical review of experimental data for the half-lives of the uranium isotopes U-238 and U-235. *Appl. Radiat. Isot.* **60**, 263–273 (2004).

47. F. Jourdan, P. R. Renne, Age calibration of the Fish Canyon sanidine $^{40}Ar/^{39}Ar$ dating standard using primary K-Ar standards. *Geochim. Cosmochim. Acta* **71**, 387–402 (2007).

48. M. O. Naumenko, K. Mezger, T. F. Nägler, I. M. Villa, High precision determination of the terrestrial ^{40}K abundance. *Geochim. Cosmochim. Acta* **122**, 353–362 (2013).

49. E. M. Niespolo, D. Rutte, A. L. Deino, P. R. Renne, Intercalibration and age of the Alder Creek sanidine ^{40}Ar/^{39}Ar standard. *Quat. Geochronol.* **39**, 205–213 (2017).

50. D. Foreman-Mackey, D. W. Hogg, D. Lang, J. Goodman, emcee: The MCMC Hammer. *Publ. Astron. Soc. Pac.* **125**, 306–312 (2013).

51. R. Cioni, L. Civetta, P. Marianella, N. Metrich, R. Santacroce, A. Sbrana, Compositional layering and Syn-eruptive mixing of a periodically refilled shallow magma chamber: The AD 79 Plinian Eruption of Vesuvius. *J. Petrol.* **36**, 739–776 (1995).

52. I. G. Smith, A Chronology for Agricola, "Mons Graupius" and Domitian's Triumph in the Chattan War. *Hist. Z. fur Alte Gesch.* **64**, 156–204 (2015).

53. D. Exiguus, "Liber de Paschate" in *Patrologiae Cursus Completus, Series Latina*, vol. 67 (J.-P. Migne, 1865).

54. H. Nesselhauf, Zwei Bronzeurkunden aus Munigua. *Madrider Mitteilungen* **1**, 142 (1960).

55. A. H. Smith, A military diploma. *J. Rom. Stud.* **16**, 95–101 (1926).

56. P. R. Renne, K. W. Min, ^{40}Ar/^{39}Ar dating of the 79 AD eruption of Vesuvius: An *ab initio* basis for imporing accuracy in ^{40}Ar/^{39}Ar geochronology. *Mineral. Mag.* **62A**, 1255–1256 (1998).

57. P. W. Reiners, R. W. Carlson, P. R. Renne, K. M. Cooper, D. E. Granger, N. M. McLean, B. Schoene, *Geochronology and Thermochronology*, (John Wiley & Sons Inc., 2017).

58. R. J. Fleck, A. T. Calvert, M. A. Coble, J. L. Wooden, K. Hodges, L. A. Hayden, M. C. van Soest, E. A. du Bray, D. A. John, Characterization of the rhyolite of Bodie Hills and ^{40}Ar/^{39}Ar intercalibration with Ar mineral standards. *Chem. Geol.* **525**, 282–302 (2019).

59. I. McDougall, T. M. Harrison, *Geochronology and Thermochronology by the ^{40}Ar/^{39}Ar Method*, (Oxford Univ. Press, 1999).

60. G. B. Dalrymple, E. C. Alexander, M. A. Lanphere, G. P. Kraker, "Irradiadion of samples for 40Ar/39Ar dating using the Geological Survey TRIGA reactor" (Geological Survey Professional Paper 1176, 1981).

61. C. Johnson, J. Lowrey, T. Alexander, E. Mace, E. B. Riley, "Measurements of emanation of Ar-37 and Ar-39 from irradiated rock and powders" (Pacific Northwest National Laboratory Report PNNL-30868, 2021).

62. N. Nica, J. Cameron, B. Singh, Nuclear data sheets for A = 36. *Nucl. Data Sheets* **113**, 1–155 (2012).
